# Judgment of emotional information expressed by prosody and semantics in patients with unipolar depression

**DOI:** 10.3389/fpsyg.2013.00461

**Published:** 2013-07-23

**Authors:** Sarah Schlipf, Anil Batra, Gudrun Walter, Christina Zeep, Dirk Wildgruber, Andreas Fallgatter, Thomas Ethofer

**Affiliations:** ^1^Department of General Psychiatry, University of TübingenTübingen, Germany; ^2^Department of Biomedical Resonance, University of TübingenTübingen, Germany

**Keywords:** depression, anhedonia, emotion, semantic content, prosody

## Abstract

It was the aim of this study to investigate the impact of major depressive disorder (MDD) on judgment of emotions expressed at the verbal (semantic content) and non-verbal (prosody) level and to assess whether evaluation of verbal content correlate with self-ratings of depression-related symptoms as assessed by Beck Depression Inventory (BDI). We presented positive, neutral, and negative words spoken in happy, neutral, and angry prosody to 23 MDD patients and 22 healthy controls (HC) matched for age, sex, and education. Participants rated the valence of semantic content or prosody on a 9-point scale. MDD patients attributed significantly less intense ratings to positive words and happy prosody than HC. For judgment of words, this difference correlated significantly with BDI scores. No such correlation was found for prosody perception. MDD patients exhibited attenuated processing of positive information which generalized across verbal and non-verbal channels. These findings indicate that MDD is characterized by impairments of positive rather than negative emotional processing, a finding which could influence future psychotherapeutic strategies as well as provide straightforward hypotheses for neuroimaging studies investigating the neurobiological correlates of impaired emotional perception in MDD.

## Introduction

Correct interpretation of emotional signals plays a key role in social interaction and can be compromised in patients with major depressive disorder (MDD). These patients display a cognitive triad pattern of thinking, consisting of negative thinking about oneself, one's life experience (and the world in general), and one's future (Wright and Beck, 1983). This triad is accompanied by maladaptive cognitive schemata (Kovacs and Beck, 1978) including enhanced attention to negative as well as impaired perception of positive information potentially leading to a biased emotion perception.

So far, several studies have been conducted to characterize this negative bias in social perception in MDD (for a review, see Garrido-Vasquez et al., 2011). Specifically, a plenitude of studies on face perception demonstrated that MDD patients tend to judge neutral or ambiguous faces as negative (Gur et al., 1992, 2002; Hale et al., 1998; Leppanen et al., 2004; Gollan et al., 2008; Douglas and Porter, 2010; Naranjo et al., 2011). In addition, attenuated perception of facial expressions depicting happiness has been demonstrated as MDD patients more frequently classified such expressions as neutral than healthy controls (Gur et al., 1992; Surguladze et al., 2004) or perceive them as less intensive (Joormann and Gotlib, 2006; Yoon et al., 2009).

In many situations, communication is conducted exclusively via the acoustic channel (e.g., telephone) and thus comprehension of social signals expressed in the auditory modality is of paramount importance to maintaining one's social network. Far less evidence, however, is available on how MDD alters perception of emotional information in the voice on the verbal (i.e., semantic meaning) and the non-verbal (i.e., prosody) level. Analogous to results in the facial domain, studies requiring the participants to make categorical judgments on acoustic emotional information revealed a negative bias specifically for ambiguous prosodic stimuli representing surprise (Kan et al., 2004) or a generally reduced ability to correctly classify prosodic information across multiple emotional categories (Uekermann et al., 2008; Peron et al., 2011). These findings converge with those obtained for recognition of non-verbal affective vocalizations (e.g., cries, screams, Naranjo et al., 2011). In a recent study in which healthy participants rated the valence of emotional prosody, a more negative evaluation of both positive and negative prosody correlated with scores obtained in a clinical rating scale for depression (Kanske and Kotz, 2011). In this study, however, only stimuli with congruent prosody valence and semantic meaning were employed. Thus, emotional information expressed at the verbal and non-verbal level could not be disambiguated. So far, only one study addressed dimensional ratings of emotional valence conveyed via visually presented words (Liu et al., 2012), demonstrating specific impairments in the processing of positive words. This concurs with findings at the neurophysiologic level indicating reduced activation within the reward system (Epstein et al., 2006) and frontal as well as limbic areas (Canli et al., 2004) in patients with MDD during perception of positive words.

Our study was designed to investigate judgment of vocal emotional information expressed by word content and prosody in MDD patients compared to a group of healthy controls (HC) matched for age, gender, and education. Specifically, we tested which of the following three competing hypothesis put forward by Garrido-Vasquez and colleagues (Garrido-Vasquez et al., 2011) can best describe the altered perception of vocal emotions in MDD:

- Hypothesis 1: There is a negative bias for emotional information in depressive patients (Beck, 1967). Based on results obtained for face processing, a general negative shift is expected which is most pronounced for ambiguous (i.e., neutral) information.
- Hypothesis 2: MDD patients exhibit an attenuation of positive vocal emotional information (Clark and Watson, 1991). According to this hypothesis, less intense ratings are expected specifically for positive stimuli.
- Hypothesis 3: A general blunting of emotions occurs in MDD (Rottenberg and Gotlib, 2004). Therefore, this hypothesis predicts less intense ratings for both positive and negative vocal emotions.

## Subjects and methods

### Participants

Twenty-three MDD patients (16 women and 7 men, mean age ± standard deviation (SD): 43.4 ± 11.2 years) and 22 HC (14 women and 8 men, 44.1 ± 10.8 years,) participated in the study. MDD patients were recruited via the psychotherapy wards (*n* = 17) and the outpatients' clinic (*n* = 6) of the University Hospital for Psychiatry and Psychotherapy Tuebingen. All MDD patients received cognitive behavioral therapy (CBT) and antidepressant medication (single medication: *n* = 12, combination therapy: *n* = 11). Antidepressant drugs included selective serotonine reuptake inhibitors (citalopram, escitalopram, fluoxetine, and sertraline), mirtazapine, venlafaxine, bupropione, quetiapine, amitriptyline, and valdoxane. MDD patients and HC were matched for educational level assessed by years in the educational system. All participants were German native speakers, right-handed according to the Edinburgh Handedness Inventory (Oldfield, 1971), and did not have any neurologic diseases. The study was approved by the Ethical Committee of the University of Tuebingen and written informed consent was obtained from all participants. All study procedures were in line with the latest version of the Declaration of Helsinki.

The diagnosis of MDD was established independently by two experienced psychiatrists. The structural clinical interview (SCID-I, First et al., 2007) was employed to exclude comorbid psychiatric diseases, such as anxiety disorders, dementia, addiction (except to nicotine), psychotic disorders, posttraumatic stress disorders, or personality disorders. Depressive symptoms were evaluated in both groups on the basis of self-ratings using the Beck Depression Inventory (BDI, German version, Hautzinger et al., 1995). Furthermore, all participants completed the self-rating of emotional intelligence test (SREIT, Schutte et al., 1998), a well-validated 33-item scale on perception, management, and utilization of emotions. MDD patients were additionally judged using the Hamilton Rating Scale for Depression (HRSD, Hamilton, 1980). All sociodemographic and clinical data are presented in Table 1. MDD patients exhibited higher depression-related symptoms as assessed by the BDI than HC (*p* < 0.001). Furthermore, MDD patients scored themselves lower in emotional intelligence (*p* < 0.001) which was expected as this patient group is typically characterized by a lower self-esteem. No other significant differences were found between the two groups.

**Table 1:** **Demographic and clinical characteristics of MDD patients (*n* = 23) and healthy controls (*n* = 22)**.

	**Age**	**Education**	**BDI**	**HRSD**	**SREIT**
	**[years]**	**[years]**	**[score]**	**[score]**	**[score]**
MDD patients	43.4 ± 11.2	15.3 ± 2.5	24.1 ± 9.1	22.0 ± 5.4	126.6 ± 13.4
HC	44.1 ± 10.8	15.6 ± 2.3	1.9 ± 2.2	n/a	112.9 ± 10.2
*p* value	ns	ns	*p* < 0.001	n/a	*p* < 0.001

MDD, Major depressive disorder; HC, Healthy controls; BDI, Beck Depression Inventory; HRSD, Hamiliton rating scale for depression; SREIT, Self-rating of emotional intelligence test; ns, Not significant; n/a, Not applicable.

### Stimuli

The stimulus set comprised 54 positive, 54 neutral, and 54 negative German adjectives which were selected from a pool of 500 words based on valence ratings obtained from 45 healthy German native speakers (Herbert et al., 2006). Raters judged the emotional valence of these words after visual presentation on a 9-point Likert scale. The selected stimuli were spoken by six professional actors (three female/three male) with happy, neutral, or angry intonation (3 semantic categories × 3 prosodic categories × 18 words = 162 stimuli) and subsequently normalized to the same peak volume (for a detailed description, see Ethofer et al., 2006a). To obtain independent ratings for classification of these acoustic stimuli as positive, neutral, or negative, all stimuli were presented to 42 healthy German native speakers who did not participate in the main experiment (mean age 28.8 years, 21 women, 21 men) in a prestudy. Volunteers were instructed to judge the valence of the emotional prosody in one session and the valence of the affective word content in the other session. The order of tasks was pseudorandomized across participants. Ratings were performed on a 9-point Likert scale (Bradley and Lang, 1994) ranging from −4 (highly negative) to +4 (highly positive). Based on these ratings, the stimuli were categorized as representing positive (valence rating > +1), neutral (valence rating between −1 and +1), or negative (valence rating < −1) information in prosody and semantic content, respectively.

### Experimental design

In the main experiment, the participants were instructed to rate the emotional valence expressed by prosody or by word content in two separate sessions comprising 81 stimuli each.

Participants were informed that there are no correct or wrong answers in this experiment and the importance of personal evaluation was emphasized. The order of stimulus presentation was randomized within sessions and the order of sessions and tasks was pseudorandomized across participants. Rating was performed on a 9-point Likert scale shown 200 ms after stimulus offset for a duration of 4 s. The cursor started in the center of the scale and the subjects could move it toward the right and left end of the scale to convey their decision. The orientation of the scale was flipped for half of the participants.

### Data analysis

We employed a three-factorial analysis of variance (ANOVA) for repeated-measures with valence (positive, neutral, negative), channel (semantics, prosody) as within- and group (HC, MDD patients) as between-subjects factor to explore general main effects and interactions of these factors. Most importantly, we compared valence ratings between MDD patients and HC using two-sample *t*-tests corrected for unequal variances separately for positive, neutral, and negative stimuli in reference to the three specific hypotheses tested in this study:

- General negative bias with most pronounced effects for ambiguous information (hypothesis 1): According to this hypothesis, positive, neutral, and negative stimuli are all rated as more negative by MDD patients than by HC. This negative bias is significantly stronger for neutral than negative stimuli.
- Attenuation of positive information (hypothesis 2): According to this hypothesis, negative biases in MDD occur during judgment of positive information. Therefore, stimuli with positive word content and happy prosody are perceived as less intense by MDD patients resulting in significantly less positive ratings of these stimuli.
- Blunting of emotional information (hypothesis 3): According to this hypothesis, less intense perception of positive and negative stimuli occurs in MDD. Therefore, a tendency toward the center is expected which results in significantly less positive ratings of positive and significantly less negative ratings of negative emotional information.

In addition, correlation analyses (Pearson's *r*) were employed to investigate whether changes in perception of emotions in word content and prosody correlate with the SREIT or depressive symptoms as assessed by the BDI. We additionally explored whether such correlations can be attributed to distinct subfactors of the SREIT (perception of emotions, management of own emotions, management of others' emotions, utilization of emotions, Schutte et al., 2009) and BDI (negative attitudes, performance difficulties, and physiological changes, Tanaka and Huba, 1984).

## Results

### Stimulus evaluation

Average ratings as obtained in the prestudy were strongly correlated with ratings of the HC group for judgment of emotional word content (*r* = 0.98) and prosody (*r* = 0.95) indicating consistent evaluation across the two independent groups of subjects.

### Judgment of emotional semantics and prosody

**Figure 1:**
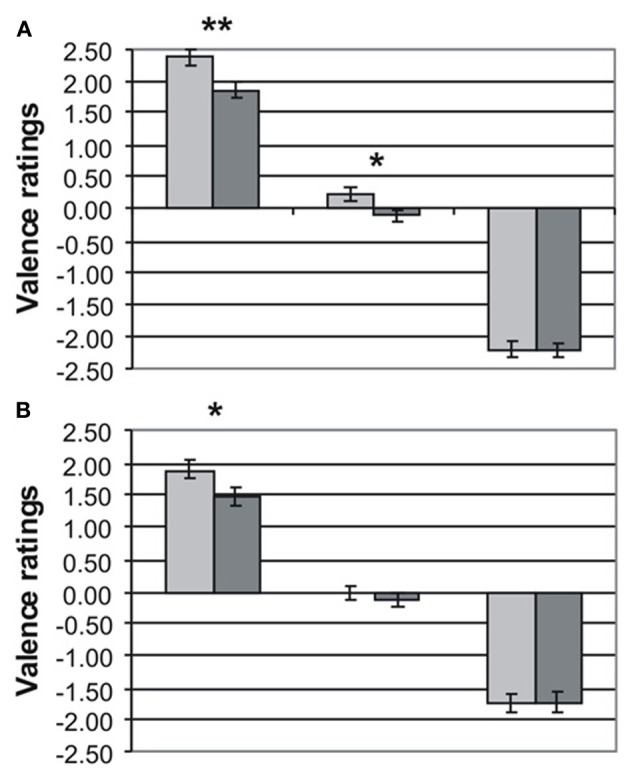
Rating (mean values ± standard errors of the mean) of emotional prosody (A) and emotional word content (B) obtained in healthy controls (light gray) and depressed patients (dark gray) for positive (left bars), neutral (center bars), and negative (right bars) emotional information. ^*^p < 0.05 ^**^p < 0.01.

Valence ratings for MDD patients (dark gray bars) and HC (light gray bars) are presented separately for emotional word content and prosody in Figures 1A,B, respectively. The three-factorial ANOVA revealed a significant main effect of valence [*F*_(2, 86)_ = 689.89, *p* < 0.001) and group [*F*_(1, 43)_ = 12.07, *p* < 0.001], but no effect of channel [*F*_(1, 43)_ = 0.41, *p* = 0.53]. The main effect of group was due to the fact that MDD patients gave significantly less intense ratings than HC to positive words [MDD: 1.80 ± 0.13 versus HC: 2.38 ± 0.12, *t*_(43)_ = 3.01, *p* < 0.01] and positive prosody [MDD: 1.48 ± 0.15 vs. HC: 1.89 ± 0.15, *t*_(43)_ = 1.88, *p* < 0.05]. Similarly, neutral words were rated more negatively by MDD patients than by HC (MDD: −0.10 ± 0.08 vs. HC: 0.21 ± 0.10, *p* < 0.05). Neutral prosody was also rated more negatively by MDD patients (MDD: −0.13 ± 0.12 vs. −0.01 ± 0.09), but this difference failed to reach significance [*t*_(43)_ = 0.78, *p* = 0.22]. Ratings of emotional information with negative valence was very similar in both groups for semantics [MDD: −2.22 ± 0.09 vs. HC: −2.20 ± 0.12, *t*_(43)_ = 0.11, *p* = 0.92] and prosody [MDD: −1.74 ± 0.16 vs. HC: −1.73 ± 0.15, *t*_(43)_ = 0.02, *p* = 0.98]. The interaction valence × channel was also significant [*F*_(2, 86)_ = 13.24, *p* < 0.001] reflecting less intense ratings in both patients and controls for emotional information in prosody than in semantics. No interactions for Valence × Group [*F*_(2, 86)_ = 2.43, *p* = 0.11], Channel × Group [*F*_(1, 43)_ = 1.43, *p* = 0.24], and Valence × Channel × Group [*F*_(2, 86)_ = 0.47, *p* = 0.86] were found.

### Correlation with BDI and sreit scores

The less intense perception of positive semantics in MDD patients versus HC correlated significantly with overall BDI scores [*r* = 0.43, *t*_(21)_ = 2.18, *p* < 0.05] and the BDI subscale for negative attitudes [*r* = 0.49, *t*_(21)_ = 2.61, *p* < 0.01]. No significant correlation of this effect was found for the BDI subscales for performance difficulties and physiological changes as well as for the SREIT and its subscales.

For judgment of positive prosody, no significant correlation with overall BDI scores was found [*r* = 0.11, *t*_(21)_ = 0.51, *p* = 0.31]. Similarly, no significant correlation of this effect was found for the BDI subscales, the SREIT or its subscales.

## Discussion

In this study, we examined evaluation of emotions expressed by prosody and word content in depressed patients. Our findings demonstrate that participants with current MDD exhibit a reduced experience of positive information which generalized across verbal and non-verbal channels. These findings are therefore in line with the hypothesis of attenuated processing of pleasant stimuli (Clark and Watson, 1991). Similar results have been obtained for rating of positive stimuli presented in other modalities, such as the taste of drinks or videos (Berenbaum and Oltmanns, 1992) as well as pictures (Sloan et al., 1997) and faces (Gur et al., 1992; Surguladze et al., 2004). This altered perception has been demonstrated to be accompanied by reduced facial reactions to Berenbaum and Oltmanns (1992); Sloan et al. (1997) and memory for positive emotions (Sloan et al., 2001). Furthermore, it has been shown that never-depressed HC exhibit a perceptual bias for positive emotions in visually perceived words (Atchley et al., 2012) as well as in conflict induced by positive words on judgment of negative faces (Hu et al., 2012) which is absent in MDD. These findings for visual and gustatory stimuli converge with our data obtained for the auditory channel and argue that MDD affects positive rather than negative emotional processing across modalities.

Interestingly, ratings for negative emotions in word content and prosody were very similar in MDD patients and HC. At first glance, these findings contradict a previous study on emotional prosody (Peron et al., 2011) which demonstrated globally impaired recognition accuracy in MDD for a broad range of emotions in both categorical judgments and intensity ratings of distinct emotional categories. However, it should be noted that our experimental design differed from the study of Peron and colleagues (Peron et al., 2011) in that the valence judgments employed in our experiment did not require any differentiation between emotions. This is particularly relevant as it is more difficult to identify the correct negative emotion among several alternatives (e.g., fear, anger, disgust, sadness) than to identify positive emotional information which typically only comprises one category (i.e., happiness, e.g., Rapport et al., 2002). Thus, judgment of negative information within the framework of such experimental designs might result in increased processing demands. As it has been shown that deficits in recognition of emotional prosody can be linked to executive functions (Uekermann et al., 2008), this could induce additional impairments. Our design avoided such biases by relying on valence ratings. It is thus unlikely that the differential effect observed in our study for positive, but not negative, emotional information is driven by general differences in executive functions.

Attenuation of perception of positive emotions in word content correlated significantly with the BDI, a self-rating scale that is strongly driven by items reflecting anhedonia (Schotte et al., 1997). These results concur with previous results on judgment of emotional words and correlation with BDI scores and other self-rating scales measuring state anhedonia (Liu et al., 2012). No significant correlation between BDI scores and attenuation of ratings of positive prosody was found. Therefore, it is possible that altered perception of positive words is a state marker, while changes in prosody perception might reflect a trait marker in MDD.

A recent study on emotion regulation revealed increased inferior frontal activity as a possible correlate of enhanced anhedonia in depressed patients (Light et al., 2011) while neuroimaging results obtained during perception of positive pictures indicates reduced activity within the reward system (Heller et al., 2009). Such hypoactivation to positive visual stimuli can be remediated by antidepressant treatment (Schaefer et al., 2006). So far, little is known about the neural structures that mediate changes in perception of affective vocal signals in MDD. Neuroimaging studies on facial emotion processing in MDD, however, revealed activation changes in the core and extended face processing system, such as fusiform face area, amygdala, and inferior frontal brain structures (for a review see Stuhrmann et al., 2011). Therefore, candidate structures for altered processing of auditory emotions in MDD might similarly depend on brain areas identified in healthy volunteers for processing of affective signals in prosody [e.g., superior temporal gyrus (Grandjean et al., 2005; Ethofer et al., 2006b), inferior frontal gyrus (Wildgruber et al., 2005; Ethofer et al., 2009), and amygdala (Wiethoff et al., 2009)] and word content [e.g., left inferior frontal cortex (Crosson et al., 2002; Ethofer et al., 2006a), left superior frontal gyrus (Cato et al., 2004; Ethofer et al., 2006a), and amygdala (Herbert et al., 2009)].

All MDD patients received antidepressive treatment including psychopharmacological medication and CBT. In the domain of face processing, several studies have shown that serotoninergic medication can change processing of emotional facial expressions (Bhagwagar et al., 2004; Harmer et al., 2004; Merens et al., 2007). Furthermore, it has been documented that neural activation to emotional faces can predict response to CBT (Costafreda et al., 2009) and it is conversely possible that CBT influenced processing of emotions in MDD patients. To our knowledge, there are no studies available that investigated whether antidepressants or CBT influence perception of vocal emotions. However, the well-known effects for faces might also extend to voices and, thus, antidepressant treatment might constitute a confounding factor. Correlation of BDI scores with altered perception of semantic content suggests that these deficits are related to the current affective state. No such correlation was found for changes in perception of prosody which suggests that impairments in understanding non-verbal emotions might be persistent and could even reflect a trait marker in MDD. However, this negative result requires careful interpretation and future studies should examine both depressed and remitted patients to make definite inference on this question.

## Conclusion

We demonstrated attenuated evaluation of positive information in MDD patients for both verbal and non-verbal vocal emotions. These findings are in line with the hypothesis emphasizing deficits in processing positive emotions (Clark and Watson, 1991). They concur with previous results obtained in other sensory channels and indicate that impaired perception of positive emotions is a general hallmark in MDD which occurs irrespective of the sensory modality. Our results further the understanding of how MDD alters perception of socially relevant cues in the voice, which is relevant for future psychotherapeutic strategies. Furthermore, these findings also provide clear hypotheses for future neuroimaging studies and thus could pave the way toward a better understanding of the neurobiological foundations of impaired perception of positive emotions in MDD.

### Conflict of interest statement

The authors declare that the research was conducted in the absence of any commercial or financial relationships that could be construed as a potential conflict of interest.
